# PROTOCOL: Evidence and gap map: studies of the effectiveness of transport sector interventions in low‐ and middle‐income countries

**DOI:** 10.1002/cl2.1136

**Published:** 2021-02-07

**Authors:** Suchi Malhotra, Howard White, Nina de la Cruz, Ashrita Saran, John Eyers, Denny John, Ella Beveridge, Nina Blöndal

**Affiliations:** ^1^ Campbell South Asia Delhi New Delhi India; ^2^ Campbell Collaboration Delhi New Delhi India; ^3^ World HealthOrganization‐Western Pacific Regional Office (WHO‐WPRO) Manila Philippines; ^4^ Independent Consultant UK; ^5^ Campbell Collaboration Ottawa Canada; ^6^ Concord Consulting Denmark

## 1 BACKGROUND^1^

### 1.1 The condition

Context: *In the Footsteps of Mr. Kurtz* Michela Wrong describes walking down the overgrown disused railway which years before had been part of a network linking DRC's copper mines to ports in Angola and South Africa. Despite new investments in the last decade—the Benguela Railway link from DRC to Angola reopened in 2018 after being closed for 34 years^2^—Africa's rail system is small compared to that in other parts of the world, and a substantial part of what there is not used (Bullock, 2009). The poor state of railway transport in Africa—and the unrealised potential of inland waterways—puts excess pressure on the fragile road transport system, so that transport costs—which are increased by uncompetitive practices—are a break on African development. While much of Africa is an extreme case, inadequate transport infrastructure is an issue across much of the developing world.

There are great disparities in the quantity and quality of infrastructure. European countries such as Denmark, Germany, Switzerland, and the UK have close to 200 km of road per 100 km^2^, and the Netherlands over 300 km per 100 km^2^. By contrast, Kenya and Indonesia have <30, Laos and Morocco <20, Tanzania and Bolivia <10, and Mauritania only 1 km per 100 km^2^.^3^ As these figures show, there is a significant backlog of transport infrastructure investment in both rural and urban areas, especially in sub‐Saharan Africa (Foster & Briceño‐Garmendia, 2010). The situation is often exacerbated by weak governance and an inadequate regulatory framework with poor enforcement which lead to high costs and defective construction.

The wellbeing of many poor people is constrained by lack of transport, which is called “transport poverty.” Lucas et al. (2016) suggest that up to 90% of the world's population are transport poor when defined as meeting at least one of the following criteria: (1) lack of available suitable transport, (2) lack of transport to necessary destinations, (3) cost of necessary transport puts household below income poverty line, (4) excessive travel time, or (5) travel conditions unsafe or unhealthy.

Benefits of better transport: better transport policies, infrastructure and services are widely believed to be important to boost sustainable, inclusive growth in low‐ and middle‐income countries (LMICs) in other regions (see, e.g., Berg et al., 2017; Abdul Quium, 2019; Simon, 2002). Transport allows people to reach jobs, education, markets, social services and engage in social and political life. Sustaining rapid economic and social development in LMICs presents a range of challenges for the transport system, a central one being to provide the capacity to accommodate increased volumes of passenger and freight traffic (Simon, 2002).

Cheap, efficient, adequate, safe, and sustainable transport services support agricultural and industrial production, inter‐ and intra‐county trade, regional integration, tourism, and the social and administrative services that are key to national and regional development. Improved transport can affect:

- Production: Transport investments can transform economies by supporting structural change, notably the shift of the population from agriculture to manufacturing and services (e.g., Calderon, 2009; Kodongo & Ojah, 2016). A study of rural roads in Bangladesh found they reduced poverty through higher agricultural production, lower input and transportation costs, and higher agricultural output prices at local village markets, as well as increasing secondary school enrolment (Khandker et al., 2009). Incorporating transaction costs into a computable general equilibrium model of Uganda, Gollin and Rogerson (2010) show that better infrastructure will stimulate agricultural production through higher farmgate prices.
- Consumption and prices: Better transport can make commodities more easily available and affordable. For example, the expansion of railways across India from the 1850s enabled market integration, which reduced prices of basic commodities such as salt.^4^ Transport‐induces changes in location of production and habitation (i.e., changes in land use) and so will affect land values. Deng et al. (2008) show that the increasing density of highways in China is a significant factor driving urban land expansion. Chalermpong (2007) estimates an elasticity of residential property prices with respect to distance from rail transit stations of −0.09.
- Access to services: Many studies show that long travel times, lack of transport services and high transportation costs are barriers to use of health services; for example, the systematic review (SR) by Kyei‐Nimakoh et al. (2017) of 160 studies of barriers to obstetric care in sub‐Saharan Africa.

These benefits are more fully elaborated in the theory of change below:

These benefits may not be realised, or be partly undermined, by the political economy context and the governance framework (Flyvbjerg, 2005; Klopp, 2012; and Alexeeva et al., 2008). Corruption and restrictive practices drive up costs, and public private partnerships (PPPs) often end up costing more than planned (Fatokun et al., 2015; Guasch et al., 2014). Transport costs are high in sub‐Saharan Africa even when the road infrastructure is adequate, due to a of lack of competition. Such considerations are an important part of the overall policy framework (Hine, 2014), but beyond the scope of this map, which is concerned with studies of effectiveness, that is, the difference transport makes to outcomes of interest.

It is thus argued that better transport is a key component to achieving several Sustainable Development Goals (SDGs): “There are a number of SDG targets directly linked to transport, including SDG 3 on health (increased road safety), SDG 7 on energy, SDG 8 on decent work and economic growth, SDG 9 on resilient infrastructure, SDG 11 on sustainable cities (access to transport and expanded public transport), SDG 12 on sustainable consumption and production (ending fossil fuel subsidies) and SDG 14 on oceans, seas and marine resources. In addition, sustainable transport will enable the implementation of nearly all the SDGs through inter‐linkage impacts. Access to sustainable transport for all should be at the forefront, including for vulnerable groups such as women, children, persons with disabilities and the elderly.”^5^

However, the presence and extent of these benefits depends on context: there is a great difference between those living in remote rural areas with little contact with the outside world and residents of a slum next to a highway in a rapidly growing city. How they interact with, transport services and policies, however, varies greatly. The impact of transport also depends on factors such as employment opportunities, access to markets and distribution of health and education facilities and other factors which may affect use of all of these. The map has to capture this full range of relevant interventions and possible policies, as well as the possible harms which may arise from transport.

Possible adverse consequences of infrastructure investments: Transport can bring disadvantages to some: displacement to make way for construction, poor road safety, higher land prices, spreading disease, air pollution, reduced accessibility on foot, moving access to jobs and goods further away and adverse cultural effects.

While transport infrastructure and services generally improve access to social services, they may have adverse effects on both health and education through the role of transport in spreading disease (the Black Death, HIV/AIDS in Africa in the 1980s and 90s, and COVID‐19 in 2020—see, e.g., Apostolopoulos & Sonmez, 2006), accidents, and a busy road through a village stopping parents sending young children to school (Jeyaranjan et al., 2010). Over 80% of road traffic deaths occur in developing countries (WHO, 2018).

Some these factors are not captured in most analyses, so there is a risk that, if adverse effects are not measured, the cost effectiveness of transport investments could be overstated and they may not produce the full range expected benefits, hence the importance of the regulatory framework. Understanding how transport policies can produce growth‐inducing effects and have social benefits, while taking into account possible adverse effects, can guide setting priorities in the strategic use of scarce resources, and setting the regulatory framework for transport investments. The challenge for transport development is thus to realise the benefits while minimizing the adverse consequences.

### 1.2 Scope of the EGM

The scope covers: (1) types of transport; (2) the policies and other actions to promote transport‐related development; (3) the outcomes of interest; (4) the population of interest; and (5) eligible study designs. Outcomes and study designs are discussed below. Here we specify (1), (2) and (4).

#### 1.2.1 Types of transport

The map will include interventions related to all kinds of transport: rail/tram, road and on foot or Motorbike/Bicycle by land, both inland waterways and international maritime transport, and air.

Road transport is the fastest surface mode of transport door‐to‐door and is most suited to short‐ to medium‐distance haul traffic. Roads provide the flexibility, the ability to provide door‐to‐door service, while providing interchange terminals for rail, water, and air transport. In many LMICs, road transport is the most dominant mode of motorised transport. For example, in the Nile region of Egypt, road transport accounts for 80% of the goods and 90% of the motorised passenger traffic in the region (Nile Basin Initiative, 2012, p. 190).

Walking is the most common means of transport in many countries, and bicycles are common for both personal and commercial use. However, the map is not about the use of a transport modality per se, but interventions which affect those choices. So a study of the effects of construction of foot paths, cycle lanes and foot bridges is included, but a study comparing say travel times or health benefits of walking, cycling and driving is not.

Railways are the most cost‐effective mode of transport for moving bulk cargo for long distances over land and are well suited to container traffic between ports and capitals. Studies have shown that rail transport costs are about 50% lower than road transport costs (Berg et al., 2017). Trams in urban areas are one of the main methods of mass passenger transit though their use has reduced considerably in the last few decades and is not common in many cities across LMICs, most notably but not only in sub‐Saharan Africa.

Maritime transport is the most dominant mode of transport for moving freight between countries with ports across the global market. Transport over sea has significant cost advantage over surface transport for dry and liquid bulk cargoes or containerised cargo but can be considerably slower than the alternatives. Where there is a steady flow of nonperishable products then this time factor matters less. Maritime transport is important as a transit route for international trade. Inland water transport on the other hand, has the advantage of being cheap, energy efficient, relatively safe, and environmentally friendly. The main type of goods and services using this transport mode comprise of agricultural produce, livestock, fish, general merchandise, and passengers. Additionally, inland ports, linked to other modes of transport connect to international markets, and handle export and import traffic of agricultural products and manufactured goods. But inland waterways are underdeveloped in LMICs, and virtually absent in sub‐Saharan Africa (International Navigation Association, 2009).

Air‐transport is the fastest mode of transport and is best suited for long‐distance movement of passengers, perishable products and high value, low‐income/low‐weight products. Air transport is linked to transport of perishable and frozen foods and precious metals. It is also important in transporting migrant labour, for example, workers from Africa and South Asia working in Gulf countries.

#### 1.2.2 The policy framework

Berg et al. (2017) categorise transport policies as falling into three broad areas: infrastructure investments, price instruments, and regulations. We use a modified version of this framework as follows:

- *Infrastructure investments* cover both new infrastructure and maintenance of existing infrastructure. Infrastructure investments entail building or maintaining new transport infrastructure (e.g., roads, railways, ports, or airports), upgrading existing links and technology, or improving transport services, such as public bus services.
- *Information and incentives* which cover a range of behaviour change interventions, which would include information (including training) for road safety, and tariffs (prices) for all forms of transport including road pricing and taxes intended to affect transport use, for example, air fuel taxes. Information includes public safety campaigns around speeding, seat belt use, and so on. Price incentives include subsidies or taxes to influence mode choice and transport behaviour (e.g., student fare reductions, tolls, parking fares, fuel taxes, and clean transport subsidies).
- *Institutional framework* which is broadly defined to include all policies and the regulations (in the case of overlap with incentives then the policy is classified as incentives). Regulations are rules to directly reduce emissions (such as fuel emission standards or driving restrictions) or to organise the transport sector including ensuring it is competitive (e.g., freight, taxis, or buses) or the construction of infrastructure.

#### 1.2.3 Population

The population are all those in all LMICs—as defined by the World Bank—in both rural and urban areas. Both national and international transport are included, but international only insofar as it affects outcomes in LMICs.

Conceptual framework of the EGM: how are the interventions expected to work.

**Figure 1:**
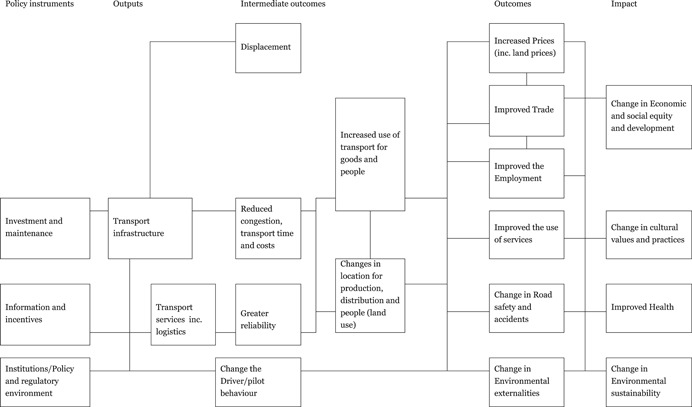
General theory of change for transport interventions

Several sources present theories of change figured for transport interventions, for example, Berg et al. (2017), Raitzer et al. (2019), and Abdul Quium (2019). Our theory of change, shown in Figure 1, draws on each of these to give a high‐level representation which applies to all our included modes of transport. The high‐level approach, cuts across all modes of transport, emphasises that there are some common causal pathways for the different modes, meaning that there are likely to be common lessons across sectors which may get overlooked by researchers and policymakers specialised in just one sector.

The theory of change shows the causal chains through which inputs are turned into outputs, intermediate and final outcomes, and higher order welfare effects (impact). On the left of the figure are the intervention areas of investment and maintenance, information and incentives, and the institutional framework (policies and regulations). As mentioned above, these effects are mediated by the political economy context and governance framework.

The availability of transport infrastructure and services affects the mediating variables through reduced travel time and greater reliability which drive location decisions for production and people, and so transport and commuting. These in turn, and together, affect a whole range of outcomes, some of which further interact: prices, internal and external trade, employment, use of services, road safety and accidents, and a range of positive and negative environmental externalities. These lead onto the changes link to changes in final welfare outcomes under the broad headings of:

- Economic and social equity and development: Effects on both economic development through trade, productivity and growth, and social development in various forms through better access. Adverse effects on displaced populations who lose their land or livelihood will also be captured here. Transport planning may mean that transport makes life harder for the poor note easier if the way in which they travel is marginalised, such as roads without pedestrian access.
- Cultural effects: The positive and negative consequences of increased mobility within and between nations. The increased mobility of the population may have effects on the culture beliefs, values, customs and norms. An example is cultural heterogeneity resulting from migration to urban areas which can result in the loss of traditional values.
- Health: Health is separated out as there are many channels through which transport can affect health, both positive (access to health services, higher income, availability of more diversified diet, etc.) and negative (road traffic injuries, air pollution, and spreading disease).
- Sustainability: Transport can have adverse effects on the environment, through impact on land use and local flora and fauna. Congestion is a growing problem, contributing to air pollution from increased traffic volumes.

This framework is used to define the categories of interventions and the outcomes along the causal chain to be shown in the map.

Why it is important to develop the EGM

Although there is no separate SDG for transport, of the 17 SDGs, seven (Goals 2, 3, 7, 9, 11, 12, and 13) include one or more targets that addresses transport, both rural and urban; and 4 (Goals 2, 3, 9, and 11) make specific reference to transport and infrastructure (United Nations, 2016). According to the Institute of Transportation and Development Policy, “this elevation of transport in SDGs recognises it as a key tool in reducing emissions, improving equity, and reducing poverty.” Analysis of these goals identifies the following key aspects of transport in the SDGs: access (urban, rural, affordable for all), road safety, fuel type/efficiency; quality, reliable, resilient, and sustainable infrastructure; regional and trans‐border transport; sustainable urban transport for all; reduce vehicle emissions/air pollution in cities; reform fossil‐fuel subsidies; rural/urban logistics, supply chain efficiency; and mitigation and adaption of climate change.

The literature on the impact of transport policies covers a variety of interventions and outcomes at different levels, such as micro, meso and macro. Due to the wide variety of interventions, mechanisms, and outcomes, a simple way to formalise the impact of transport policies is to how these policies affect the welfare of individuals or groups, improve regulation and infrastructure, would be quite useful. At the same time, as explained above, the expansion of transport in LMICs has brought out both positive and negative effects.

The purpose of this map is to document all relevant studies, from all sectors, which analyse the effects of transport interventions. The nearest study to what we will do is the ADB review of transport impact evaluations by Raitzer et al. (2019). But that review was not systematic and more restricted to analysis by economists. We have a broader disciplinary scope, most notably bringing in the relevant engineering literature.

Existing EGMs and/or relevant SRs

A map of evidence maps conducted in LMICs identified no EGM conducted around transportation (Phillips et al., 2017). The lack of such a map was the rationale for starting this map. There is an on‐going global map of road safety (Mohan et al., 2020).

Table 1 lists some reviews of transport sector interventions. These are illustrative of the sort of topics, which may be covered; they have not been screened to determine whether they include primary studies from LMICs.

**Table 1:** Systematic review of transport systems^a^

Interventions	Roads, cycle paths, and pavements/walkways	Railways	Shipping and waterways
Investments and maintenance	Egan et al. (2011) New roads and human health	Havârneanu et al. (2015) A systematic review of the literature on safety measures to prevent railway suicides and trespassing accidents	
	Benítez‐López et al. (2010) The impacts of roads and other infrastructure on mammal and bird populations: A meta‐analysis	
	Cavil et al. (2008) Economic analyses of transport infrastructure and policies including health effects related to cycling and walking	Bastiaanssen et al. (2020) Does transport help people to gain employment? A systematic review and meta‐analysis of the empirical evidence
	Hine et al. (2015) The poverty reduction impact of rural roads: a systematic review; and Hine et al. (2019). Evidence on impact of rural roads on poverty and economic development	Kasraian et al. (2016)
		Long‐term impacts of transport infrastructure networks on land‐use change: an international review of empirical studies
Information and incentives	Ogilvie et al. (2004) Promoting walking and cycling as an alternative to using cars: Systematic review		
Policy and regulatory environment	Heath et al. (2006) The effectiveness of urban design and land use and transport policies and practices to increase physical activity: A systematic review		Vieira et al. (2014) Governance, governance models and port performance: A systematic review

^a^ Air transport excluded as no relevant reviews were found. A reviewer mentioned an on‐going review of Air Transport in Low‐ and Middle‐Income Countries by Foster and Bofinger, which we will include if we locate it.

## 2 OBJECTIVES

The aim of the EGM is to identify, map and describe existing evidence on the effects of transport sector interventions related to all means of transport (roads, paths, cycle lanes, bridges, railways, ports, shipping and inland waterways, and air transport). These interventions are classified as shown in the theory of change (Figure 1), that is infrastructure and maintenance, information and incentives, and institutional framework (including regulations) transportation in LMICs. The primary outcomes of this EGM are also shown in Figure 1 and include transport infrastructure, economic and social development, safety, environmental and environmental health, and economic and equity outcomes.

Specifically, the objectives of the map are to:

a) Develop a clear framework of interventions and outcomes related to effects of transport in LMICs
b) Map available SRs and primary studies of the social and economic effects of interventions aimed at improving transportation in LMICs in this framework, with an overview provided in a summary report.
c) Provide database entries of included studies, which summarise the intervention, context, study design, and main findings.

The output of the project will be an online, interactive evidence and gap map (EGM) for all forms of transport, such as roads, railways (including mass transportation and bulk transport of energy and related commodities), civil aviation, ports and inland water transport, and urban transport.

## 3 METHODOLOGY

### 3.1 Defining EGMs

While SRs aim to identify, assess and summarise research findings from studies on a (narrow) research question, the objective of EGMs is to provide a picture of the completeness of existing research literature on a given topic. As such, EGMs have a broader scope than SRs, and SRs go further than EGMs in processing the contents of the identified research. Another important difference between EGMs and SRs is how they are disseminated. SRs are disseminated as research reports or journal articles, where the answer to the research question is the key issue for readers. EGMs can also be disseminated as a report or an article, but the more user‐friendly EGMs display its results in an interactive matrix. Identified studies are plotted in the matrix, so that the user can find evidence, or lack thereof, for his or her particular topic of interest, at a glance. EGM are global public goods that attempt to democratise high quality research evidence for policy makers, practitioners, and public and research funders. The EGM presented here includes evidence from impact evaluations and SRs.

### 3.2 EGM framework

#### 3.2.1 Population

The target population are populations living in LMICs. Populations subgroups of interest, which are informed by PROGRESS+ equity criteria (O'Neill et al., 2013) include: rural/urban, women, disability, older population, ethnicity, poorer populations, region, and country. These subgroups will be added to the map as filters.

#### 3.2.2 Intervention

The EGM will include any intervention aiming to construct, improve, maintain or affect the use of transportation in LMICs in the above categories of modes of transport. Broadly, there are three policies that have contributed to improving transport networks; infrastructure investments, price instruments (which we broaden to incentives), and regulations (Berg et al., 2017). The infrastructure investments entail building new transport infrastructure (e.g., roads, railways, ports, or airports), upgrading existing links and technology, or improving transport services. The incentives include subsidies or taxes to influence mode choice and transport behaviour (e.g., student fare reductions, tolls, parking fares, fuel taxes, and clean transport subsidies). The regulations include rules to directly reduce emissions (such as fuel emission standards or driving restrictions) or to organise the transport sector (e.g., freight, taxis, or buses) or the construction of infrastructure. Some policy interventions may affect supply, such as infrastructure investments, whereas others target demand, as do subsidies for transport.

We reframe Berg et al.'s three categories (infrastructure, prices, and regulations) a bit more broadly as infrastructure, incentives, and institutions. So, the intervention categories are each mode of transport, and the subcategories in each case those just mentioned, that is, infrastructure, information, and incentives (which is broader than price mechanism) and institutions (which is broader than regulation). Table 2 shows the resulting set of intervention categories.

**Table 2:** Intervention categories and subcategories

Category	Subcategories	Examples
Road, paths, and footbridges	Infrastructure	Construction and upgrading of roads, and highways
		Infrastructure maintenance
	Incentives	Road pricing and tolls
		Subsidies and taxes
	Institutions (including regulations)	Road legislation and agencies
		Vehicle and driving regulations
		Public private partnership (PPP)
Rail and trams	Infrastructure	Construction and upgrading
		Maintenance
	Incentives	Pricing structure
		Subsidies to rail operators
	Institutions (including regulations)	Regulatory framework
		Public private partnership (PPP)
		Nationalisation/privatisation
Ports, shipping, and waterways	Infrastructure	Port and inland waterway construction and rehabilitation including modernization
		Maintenance
	Incentives	Tolls and other charges
		Taxes and subsidies
	Institutions (including regulations)	Port authorities
Civil aviation	Infrastructure	Airports
	Incentives	Taxes and subsidies
	Institutions (including regulations)	Airport authorities

#### 3.2.3 Outcomes

The outcomes are listed in outcome domains ordered along the causal chain (Table 3). Each domain has a number of subdomains. The map covers positive and adverse outcomes, and sufficient scope to capture unintended outcomes.

**Table 3:** EGM outcomes

Domain	Subdomain
Transport infrastructure, services and use	Infrastructure quantity
	Infrastructure quality (inc. safety assessment)
	Infrastructure services
	Transport time or costs (inc. congestion and VOC)
	Market access
	Transport modality (inc. car ownership)
	Safe practices
Economic impact	Household income and poverty
	Economic development
	Employment and migration
	Trade and tourism
	Location (land use) and prices
	Displacement
Health and education	Access to health facilities
	Health outcomes
	Access to education facilities
	Education outcomes
Culture	Values, language, and social cohesion
	Cultural heritage
	Cultural diversity
Environment	Air quality
	Noise pollution
	Habitat destruction
Economic and equity analysis	Cost effectiveness or CBA
	Gender equity
	Transport equity 6

6 Transportation equity or justice usually refers to the fairness with which the impacts of transportation such as benefits and costs are distributed. Horizontal equity, also called fairness and egalitarianism, is concerned with the distribution of impacts between individuals and groups considered equal in ability and need; vertical equity is concerned with the distribution of impacts between individuals and groups that differ in abilities and needs, for example, by income or social class (also called social justice, environmental justice, and social inclusion) or in transportation ability and need otherwise known as universal design (Litman, 2018).

While, cost benefit/benefit‐cost analysis is an exercise to determine the social welfare effects of transport sector interventions in comparison to costs, economic impact analysis is an exercise to determine how a transport intervention project or policy affects the amount and type of economic activity in a region.

In addition, if included studies report costs related to the costs of transport infrastructure, their cost‐effectiveness or cost‐benefits, and/or economic impact and/or transport justice/equity these will be reported as well.

### 3.3 Criteria for including and excluding studies

#### 3.3.1 Types of study designs

There are many policy‐relevant areas of research on transport, including barriers to access, costs and governance arrangements. Qualitative data and studies can play an important role on complementing impact evaluations; see White (2011) on mixed methods impact evaluations in infrastructure. However, this transport map is a map of effectiveness studies, and so excludes qualitative studies. The rationale is the comparative lack of measures of impact on outcomes of interest using impact evaluation methods. But there is a growing literature. Making this literature discoverable and accessible will be the main contribution of this map.

The map is timely because the number of impact evaluations has been growing across development sectors. By impact evaluations we mean studies which assess the difference an intervention makes to outcomes, employing a technique which handles the possible endogeneity of exposure to the intervention. This endogeneity is at the heart of discussions on transport and development. In the *Handbook of Transport and Development* (in which the cases are mostly from developed countries), the authors state in the introduction that “Often it seems that development follows the transport infrastructure… But the causality is rarely in one direction and often the development form helps shape the transport infrastructure investments” (Hickman et al., 2015, p. 3).

This EGM will include impact evaluations and SRs of the effectiveness of transport sector interventions.

The impact evaluations will include:

- Experimental designs: randomized controlled trials (RCTs) and natural experiments
- Nonexperimental designs: (i) quasi experimental designs using statistical methods to create a comparison group such as propensity score matching and regression discontinuity, (ii) regression‐based designs such as instrumental variables and Heckmann sample selection models; and (iii) other studies with a comparison group. Before versus after studies with no comparison group will not be included.
- Regression designs which control for confounding variables.

#### 3.3.2 Treatment of qualitative research

We will not include qualitative research.

#### 3.3.3 Types of settings

All included impact evaluations must have been conducted in LMICs as defined by the World Bank. SRs containing evidence only from high‐income countries will be excluded.

For civil aviation, we will exclude studies focusing on passenger transport.

#### 3.3.4 Status of studies

We will search for and include completed and on‐going studies. We will not exclude any studies based on language or publication status or publication date.

### 3.4 Search strategy and status of studies

We will use the following strategies to identify completed and on‐going potential studies:

Database: EconLit, Embase (Ovid), MEDLINE (Ovid), CAB Global Health, CAB Abstracts, Cochrane Library, ERIC (Proquest), Social Sciences Citation Index (Web of Science), Transport Database (Ovid) (or https://trid.trb.org/), Proquest Dissertations & Theses, WHO Global Health Library, Ebsco Discovery.

Organisational searches will include: 3ie impact evaluation repository, Asian Development Bank (ADB), African Development Bank, Inter‐American Development Bank, Department of International Development (DFID), US Agency for International Development, World Bank (DIME, Impact Evaluations), IFPRI, IPA, Transport Research Institute, Napier University UK, European Conference of Transport Research Institutes, International Rail Research Board (IRRB), Asian Institute of Transport Development, Institute of Transport Research, European Conference of Transport Research Institutes. We will contact a selection of these agencies for details of on‐going research.

Bibliographic searches: We will screen the SRs to locate additional primary studies.

We will also conduct bibliographic back‐referencing of reference lists of all included SRs to identify additional primary studies and SRs.

In addition, we will identify developing country studies in the on‐going map of road safety interventions.

In the case of on‐going studies we will search for the completed study and include the study as on‐going if no completed study is found.

Hand search of journals: we will identify key journals from the search results and hand search the contents of the last five years of up to 12 of these journals.

Appendix A presents an example of the search strings used for publication databases and search engines, with terms for interventions, regions, and methodologies.

### 3.5 Screening and selection of studies

We will use EPPI reviewer to assess studies for inclusion at both the title/abstract and full‐text screening stages. Two researchers will screen each title/abstract and each full‐text. Any disagreements on inclusion will be resolved through discussion.

### 3.6 Data extraction, coding, and management

For impact evaluations, we will use a standardised data extraction form to extract descriptive data from all studies meeting our inclusion criteria. Data extracted from each study will include bibliographic details, intervention types and descriptions, outcome types and descriptions, study design, context/geographical information, details on the comparison group, and implementation details.

A full list of data to be extracted is described in the coding tool in Annexure C (Data extraction template); this tool will be piloted to ensure consistency in coding and resolve any issues or ambiguities. Two researchers will conduct the data extraction for each study; however, all coders will be trained on the tool before starting and a sample will be double coded to check for consistency.

For SRs, a modified version of the tool will be developed for the data extraction.

### 3.7 Quality appraisal

All SRs will be appraised for quality using the AMSTAR2 or ROBIS tool. Critical appraisal will be completed separately by two team members.

We will not be critically appraising the quality of the included impact evaluations but will collect data on study design.

## 4 ANALYSIS AND PRESENTATION

### 4.1 Unit of analyses

Where multiple papers exist on the same study (e.g., working paper and a published version), the most recent open access version will be included in the EGM. If the versions report on different outcomes, an older version will be included for the outcomes not covered in later versions.

### 4.2 Planned analyses

The matrix and filters are described in Appendix A (Evidence matrix). In brief, the matrix will display interventions categories, intervention subcategories, against outcomes for each mode of transport. It will be searchable by filters such as infrastructure, incentives, institutions (including regulations), geography (urban, rural, country, region), study design (RCT, non‐RCT, cost‐effectiveness, cost‐benefit, economic impact, equity analysis), and study status (completed, ongoing). The report will include descriptions of the evidence base according to these categories and present a global map, tables and figures presenting descriptive information about these characteristics. The report will present separately evidence from primary research (impact evaluations) and synthesis (SRs).

### 4.3 Presentation

The matrix and filters described above and in Appendix A. In brief, the matrix will display interventions (road, rail and trams, ports, shipping and waterways, and civil aviation), subcategory (infrastructure, incentives, institutions (including regulations)), against outcomes for each mode of transport. It will searchable by various filters including intervention, study design, study method, country and global region, and location (rural, urban).

## 5 STAKEHOLDER ENGAGEMENT

We have engaged stakeholders on the evidence matrix at various organisation who work on transport sector interventions. These include TERI University, Department of Civil Engineering, IIT‐Delhi, and Independent Council for Road Safety International (ICORSI).

Once we have a draft of the map we will reach out to the World Bank, ADB, and African Development Bank as well as transport economics experts in a range of low‐income countries including Africa. It should also reach out to key leading global university transport research centres including University of Sydney, University of Leeds and LET, University of Lyon. Our previous experience is that consulting those unfamiliar with maps without a product to show them can be a mutually frustrating process.

## ROLES AND RESPONSIBILITIES

- *Content expertise:*
  Nina Blöndal has conducted several impact evaluations of transport interventions and authored a chapter on transport impact evaluation for the ADB Guidebook. Dr. Howard White co‐edited a special issue of the Journal of Development Effectiveness on infrastructure impact evaluations including contributing a paper on mixed methods in infrastructure studies.

- *Systematic review method expertise*:
  All authors are experienced systematic reviewers, which means that they are proficient in conducting various processes in an EGM, such as screening, quality assessment and coding. Howard White will provide technical support for the conducting the review.
- *EGM methods expertise*:
  Howard White as CEO provides technical and strategic support for the development of EGM in Campbell library. All team members have previous experience in systematic review methodology, including search, data collection, statistical analysis, theory‐based synthesis, which mean they are proficient in carrying out the various processes in an EGM, such as search, eligibility screening, quality assessment, and coding.
- *Information retrieval expertise*:
  The authors will be supported by information retrieval specialist, Dr. John Eyers, on an as‐needed basis. John Eyers is a trained information retrieval specialist and has experience of supporting over 50 systematic maps and reviews in social sciences areas.

## SOURCES OF SUPPORT

This EGM is supported by the UK Department of International Development (DFID) under its support for the Centre for Excellence for Development Impact and Learning (CEDIL).

## DECLARATIONS OF INTEREST

No conflicts of interest.

## PRELIMINARY TIMEFRAME

The draft map will be ready in January 2021, and the revised version by March 2021.

## PLANS FOR UPDATING THE EGM

We plan to update the map (or support others in doing so) when sufficient further studies and resources become available.

## Footnotes

1 This section draws on Berg et al. (2017).

2
https://www.railjournal.com/africa/angola-drc-rail-link-restored-after-34-years/.

3
https://knoema.com/atlas/ranks/Road-density.

4 A brief overview of studies is given in https://blogs.lse.ac.uk/southasia/2013/04/29/railways-and-indian-economic-development/.

5
https://sustainabledevelopment.un.org/index.php?page=view&type=20000&nr=802&menu=2993.

## APPENDIX A FRAMEWORK

**Intervention categories and subcategories**

**Table:**

Category	Subcategories	Examples
Roads and pathways (including cycle paths)	Infrastructure	Construction and upgrading of roads, and highways
		Infrastructure maintenance
	Incentives	Road pricing and tolls
		Subsidies and taxes
	Institutions (including regulations)	Road legislation and agencies
		Vehicle and driving regulations
		Public private partnership (PPP)
Rail and trams	Infrastructure	Construction and upgrading
		Maintenance
	Incentives	Pricing structure
		Subsidies to rail operators
	Institutions (including regulations)	Regulatory framework
		Public private partnership (PPP)
		Nationalisation/privatisation
Ports, shipping, and waterways	Infrastructure	Port and inland waterway construction and rehabilitation including modernization
		Maintenance
	Incentives	Tolls and other charges
		Taxes and subsidies
	Institutions (including regulations)	Port authorities
Civil aviation	Infrastructure	Airports
	Incentives	Taxes and subsidies
	Institutions (including regulations)	Airport authorities

*Outcomes*

**Table:**

Domain	Subdomain
Transport infrastructure, services and use	Infrastructure quantity
	Infrastructure quality (inc. safety assessment)
	Infrastructure services
	Transport time or costs (inc. congestion and VOC)
	Market access
	Transport modality (inc. car ownership)
	Safe practices
Economic Impact	Household income and poverty
	Economic Development
	Employment and migration
	Trade and tourism
	Location (land use) and prices
	Displacement
Health and education	Access to health facilities
	Health outcomes
	Access to education facilities
	Education outcomes
Culture	Values, language and social cohesion
	Cultural heritage
	Cultural diversity
Environment	Air quality
	Noise pollution
	Habitat destruction
Economic and equity analysis	Cost effectiveness or CBA
	Gender equity
	Transport equity 7

7 Transportation equity or justice usually refers to the fairness with which the impacts of transportation such as benefits and costs are distributed. Horizontal equity, also called fairness and egalitarianism, is concerned with the distribution of impacts between individuals and groups considered equal in ability and need; vertical equity is concerned with the distribution of impacts between individuals and groups that differ in abilities and needs, for example, by income or social class (also called social justice, environmental justice and social inclusion) or in transportation ability and need otherwise known as universal design (Litman, 2018).

## APPENDIX B SEARCH TERMS

**Intervention search term**

- DE "TRANSPORTATION" OR DE "AIR travel" OR DE "AUTOMOTIVE transportation" OR DE "BUS transportation" OR DE "CARRIAGES & carts" OR DE "CARRIERS" OR DE "CHOICE of transportation" OR DE "COMMUTING" OR DE "DEEP sea passenger transportation" OR DE "DELIVERY of goods" OR DE "DRAYAGE" OR DE "EMERGENCY transportation" OR DE "EMPLOYER‐sponsored transportation" OR DE "FERRIES" OR DE "FERRY routes" OR DE "FREIGHT & freightage" OR DE "GROUND passenger transportation" OR DE "HARBORS" OR DE "HIGH speed ground transportation" OR DE "INTERNATIONAL transit" OR DE "OCEAN travel" OR DE "PASSES (Transportation)" OR DE "PUBLIC transit" OR DE "RAILROAD travel" OR DE "RAILROADS" OR DE "ROADS" OR DE "ROUTE surveying" OR DE "RURAL transportation" OR DE "SHIPPING (Water transportation)" OR DE "SHUTTLE services" OR DE "SUSTAINABLE transportation" OR DE "TAXI service" OR DE "TRANSPORTATION demand management" OR DE "TRANSPORTATION management system" OR DE "TRANSPORTATION of school children" OR DE "URBAN transportation" OR DE "VEHICLES" OR DE "WAGON trains" OR DE "WATERWAYS" OR DE "FINANCING of transportation" OR DE "PUBLIC transit commissions" OR DE "TRANSPORTATION accidents" OR DE "TRANSPORTATION agencies" OR DE "TRANSPORTATION departments" OR DE "TRANSPORTATION industry" OR DE "TRANSPORTATION laws" OR DE "TRANSPORTATION policy"
- (DE "inland transport" OR DE "international transport" OR DE "long distance transport" OR DE "air transport" OR DE "rail transport" OR DE "refrigerated transport" OR DE "road transport" OR DE "bus transport" OR DE "airports" OR DE "railways" OR DE "roads" OR DE "transport costs" OR DE "transporting quality" OR DE "water transport" OR DE "waterways" OR DE "transport") OR DE “rural transport”
- TI ((Infrastructur* OR maintenance or maintain* OR repair* OR construction OR upgrade OR upgrading]) N6 (road* OR rail* OR tram* OR port OR ports OR ship* OR ships OR shipping OR waterway* OR aviation OR aircraft* OR "mass transport*" OR subway* OR transportation OR busway OR highway OR taxi* OR auto* OR "public trans*" OR "commuter trans*" OR "mass transit" OR "commuter train*" OR "passenger trans*" OR "passenger train*" OR trucks OR trucking OR freight OR lorry OR lorries OR vehicles))) OR AB ((Infrastructur* OR maintenance or maintain*) N6 (road* OR rail* OR tram* OR port OR ports OR ship* OR ships OR shipping OR waterway* OR aviation OR aircraft* OR "mass transport*" OR subway*)) OR SU ((Infrastructur* OR maintenance or maintain*) N6 (road* OR rail* OR tram* OR port OR ports OR ship* OR ships OR shipping OR waterway* OR aviation OR aircraft* OR "mass transport*" OR subway*))
- TI ((Incentiv* OR price OR prices OR pricing OR tariff* OR toll*) N6 (road* OR rail* OR tram* OR port OR ports OR ship* OR ships OR shipping OR waterway* OR aviation OR aircraft* OR "mass transport*" OR subway*)) OR AB ((Incentiv* OR price OR prices OR pricing OR tariff* OR toll*) N6 (road* OR rail* OR tram* OR port OR ports OR ship* OR ships OR shipping OR waterway* OR aviation OR aircraft* OR "mass transport*" OR subway*)) OR SU ((Incentiv* OR price OR prices OR pricing OR tariff* OR toll*) N6 (road* OR rail* OR tram* OR port OR ports OR ship* OR ships OR shipping OR waterway* OR aviation OR aircraft* OR "mass transport*" OR subway*))
- TI ((institution* OR organiz* OR organis* OR regulat*) N6 (road* OR rail* OR tram* OR port OR ports OR ship* OR ships OR shipping OR waterway* OR aviation OR aircraft* OR "mass transport*" OR subway*)) OR AB ((institution* OR organiz* OR organis* OR regulat* * OR policy OR policies OR law OR laws OR legistlat* OR agencies OR "public private partnership" OR privatization OR privatisation OR nationalization OR nationalisation) N6 (road* OR rail* OR tram* OR port OR ports OR ship* OR ships OR shipping OR waterway* OR aviation OR aircraft* OR "mass transport*" OR subway*)) OR SU ((institution* OR organiz* OR organis* OR regulat*) N6 (road* OR rail* OR tram* OR port OR ports OR ship* OR ships OR shipping OR waterway* OR aviation OR aircraft* OR "mass transport*" OR subway*))
- TI (public private partnership OR PPP) OR (transport) N6 (access* OR services) OR (HDM‐4) OR (road OR bridge OR congestion OR emission OR planning) N6 (toll OR charge OR tax)
  **Study design search terms**
- TI (((quantitativ* N5 synthes*) OR "mixed method*" or mixed‐method*)) OR AB (((quantitativ* N5 synthes*) OR "mixed method*" or mixed‐method*)) OR SU (((quantitativ* N5 synthes*) OR "mixed method*" or mixed‐method*))
- TI ((random$ or RCT or "double difference" or "regression discontinuity" or "propensity score" or matching or "comparison group" or "control group" or "instrumental variable*" or heckmann)) OR AB ((random$ or RCT or "double difference" or "regression discontinuity" or "propensity score" or matching or "comparison group" or "control group" or "instrumental variable*" or heckmann)) OR SU ((random$ or RCT or "double difference" or "regression discontinuity" or "propensity score" or matching or "comparison group" or "control group" or "instrumental variable*" or heckmann))
- TI (("meta regression" or "meta synth*" or "meta‐synth*" or "meta analy*" or metaanaly* or meta‐analy* or metanaly* or "metaregression" or meta‐regression or "methodologic* overview" or "pool* analys*" or "pool* data" or "quantitative* overview" or "research integration")) OR AB (("meta regression" or "meta synth*" or "meta‐synth*" or "meta analy*" or metaanaly* or meta‐analy* or metanaly* or "metaregression" or meta‐regression or "methodologic* overview" or "pool* analys*" or "pool* data" or "quantitative* overview" or "research integration")) OR SU (("meta regression" or "meta synth*" or "meta‐synth*" or "meta analy*" or metaanaly* or meta‐analy* or metanaly* or "metaregression" or meta‐regression or "methodologic* overview" or "pool* analys*" or "pool* data" or "quantitative* overview" or "research integration"))
- TI (((systematic* or synthes*) N3 (research or evaluation* or finding* or thematic* or report or descriptive or explanatory or narrative or meta* or review*)) or (map N3 (evidence or gap))) OR AB (((systematic* or synthes*) N3 (research or evaluation* or finding* or thematic* or report or descriptive or explanatory or narrative or meta* or review*)) or (map N3 (evidence or gap))) OR SU (((systematic* or synthes*) N3 (research or evaluation* or finding* or thematic* or report or descriptive or explanatory or narrative or meta* or review*)) or (map N3 (evidence or gap)))

**LMIC search terms‐**

- TI (("transitional countr*" or "emerging econom*" or "global south")) OR AB (("transitional countr*" or "emerging econom*" or "global south"))
- TI ((lmic or lmics or "third world" or "lami countr*")) OR AB ((lmic or lmics or "third world" or "lami countr*"))
- TI (low N1 middle N1 countr*) OR AB (low N1 middle N1 countr*)
- TI ((low* N1 (gdp or gnp or "gross domestic" or "gross national"))) OR AB ((low* N1 (gdp or gnp or "gross domestic" or "gross national")))
- TI (((developing or "less* developed" or "least developed" or "under developed" or underdeveloped or "middle income" or "low* income" or underserved or "under served" or deprived or poor* or "resource limited" or "resource constrained") N1 (economy or economies))) OR AB (((developing or "less* developed" or "least developed" or "under developed" or underdeveloped or "middle income" or "low* income" or underserved or "under served" or deprived or poor* or "resource limited" or "resource constrained") N1 (economy or economies)))
- TI (((developing or "less* developed" or "least developed" or "under developed" or underdeveloped or "middle income" or "low* income" or underserved or "under served" or deprived or poor* or "resource limited" or "resource constrained") N1 (countr* or nation? or population? or world or state*))) OR AB (((developing or "less* developed" or "least developed" or "under developed" or underdeveloped or "middle income" or "low* income" or underserved or "under served" or deprived or poor* or "resource limited" or "resource constrained") N1 (countr* or nation? or population? or world or state*)))
- TI ((Afghanistan or Albania or Algeria or Angola or Argentina or Armenia or Armenian or Azerbaijan or Bangladesh or Benin or Byelarus or Byelorussian or Belarus or Belorussian or Belorussia or Belize or Bhutan or Bolivia or Bosnia or Herzegovina or Hercegovina or Botswana or Brazil or Bulgaria or "Burkina Faso" or "Burkina Fasso" or "Upper Volta" or Burundi or Urundi or Cambodia or "Khmer Republic" or Kampuchea or Cameroon or Cameroons or Cameron or Camerons or "Cape Verde" or "Central African Republic" or Chad or China or Colombia or Comoros or "Comoro Islands" or Comores or Mayotte or Congo or Zaire or "Costa Rica" or "Cote d'Ivoire" or "Ivory Coast" or Cuba or Djibouti or "French Somaliland" or Dominica or "Dominican Republic" or "East Timor" or "East Timur" or "Timor Leste" or Ecuador or Egypt or "United Arab Republic" or "El Salvador" or Eritrea or Ethiopia or Fiji or Gabon or "Gabonese Republic" or Gambia or Gaza or "Georgia Republic" or "Georgian Republic" or Ghana or Grenada or Guatemala or Guinea or Guiana or Guyana or Haiti or Honduras or India or Maldives or Indonesia or Iran or Iraq or Jamaica or Jordan or Kazakhstan or Kazakh or Kenya or Kiribati or Korea or Kosovo or Kyrgyzstan or Kirghizia or "Kyrgyz Republic" or Kirghiz or Kirgizstan or "Lao PDR" or Laos or Lebanon or Lesotho or Basutoland or Liberia or Libya or Macedonia or Madagascar or "Malagasy Republic" or Malaysia or Malaya or Malay or Sabah or Sarawak or Malawi or Mali or "Marshall Islands" or Mauritania or Mauritius or "Agalega Islands" or Mexico or Micronesia or "Middle East" or Moldova or Moldovia or Moldovian or Mongolia or Montenegro or Morocco or Ifni or Mozambique or Myanmar or Myanma or Burma or Namibia or Nepal or "Netherlands Antilles" or Nicaragua or Niger or Nigeria or Muscat or Pakistan or Palau or Palestine or Panama or Paraguay or Peru or Philippines or Philipines or Phillipines or Phillippines or "Papua New Guinea" or Romania or Rumania or Roumania or Rwanda or Ruanda or "Saint Lucia" or "St Lucia" or "Saint Vincent" or "St Vincent" or Grenadines or Samoa or "Samoan Islands" or "Navigator Island" or "Navigator Islands" or "Sao Tome" or Senegal or Serbia or Montenegro or Seychelles or "Sierra Leone" or "Sri Lanka" or "Solomon Islands" or Somalia or Sudan or Suriname or Surinam or Swaziland or Eswatini or "South Africa" or Syria or Tajikistan or Tadzhikistan or Tadjikistan or Tadzhik or Tanzania or Thailand or Togo or "Togolese Republic" or Tonga or Tunisia or Turkey or Turkmenistan or Turkmen or Uganda or Ukraine or Uzbekistan or Uzbek or Vanuatu or "New Hebrides" or Venezuela or Vietnam or "Viet Nam" or "West Bank" or Yemen or Zambia or Zimbabwe)) OR AB ((Afghanistan or Albania or Algeria or Angola or Argentina or Armenia or Armenian or Azerbaijan or Bangladesh or Benin or Byelarus or Byelorussian or Belarus or Belorussian or Belorussia or Belize or Bhutan or Bolivia or Bosnia or Herzegovina or Hercegovina or Botswana or Brazil or Bulgaria or "Burkina Faso" or "Burkina Fasso" or "Upper Volta" or Burundi or Urundi or Cambodia or "Khmer Republic" or Kampuchea or Cameroon or Cameroons or Cameron or Camerons or "Cape Verde" or "Central African Republic" or Chad or China or Colombia or Comoros or "Comoro Islands" or Comores or Mayotte or Congo or Zaire or "Costa Rica" or "Cote d'Ivoire" or "Ivory Coast" or Cuba or Djibouti or "French Somaliland" or Dominica or "Dominican Republic" or "East Timor" or "East Timur" or "Timor Leste" or Ecuador or Egypt or "United Arab Republic" or "El Salvador" or Eritrea or Ethiopia or Fiji or Gabon or "Gabonese Republic" or Gambia or Gaza or "Georgia Republic" or "Georgian Republic" or Ghana or Grenada or Guatemala or Guinea or Guiana or Guyana or Haiti or Honduras or India or Maldives or Indonesia or Iran or Iraq or Jamaica or Jordan or Kazakhstan or Kazakh or Kenya or Kiribati or Korea or Kosovo or Kyrgyzstan or Kirghizia or "Kyrgyz Republic" or Kirghiz or Kirgizstan or "Lao PDR" or Laos or Lebanon or Lesotho or Basutoland or Liberia or Libya or Macedonia or Madagascar or "Malagasy Republic" or Malaysia or Malaya or Malay or Sabah or Sarawak or Malawi or Mali or "Marshall Islands" or Mauritania or Mauritius or "Agalega Islands" or Mexico or Micronesia or "Middle East" or Moldova or Moldovia or Moldovian or Mongolia or Montenegro or Morocco or Ifni or Mozambique or Myanmar or Myanma or Burma or Namibia or Nepal or "Netherlands Antilles" or Nicaragua or Niger or Nigeria or Muscat or Pakistan or Palau or Palestine or Panama or Paraguay or Peru or Philippines or Philipines or Phillipines or Phillippines or "Papua New Guinea" or Romania or Rumania or Roumania or Rwanda or Ruanda or "Saint Lucia" or "St Lucia" or "Saint Vincent" or "St Vincent" or Grenadines or Samoa or "Samoan Islands" or "Navigator Island" or "Navigator Islands" or "Sao Tome" or Senegal or Serbia or Montenegro or Seychelles or "Sierra Leone" or "Sri Lanka" or "Solomon Islands" or Somalia or Sudan or Suriname or Surinam or Swaziland or Eswatini or "South Africa" or Syria or Tajikistan or Tadzhikistan or Tadjikistan or Tadzhik or Tanzania or Thailand or Togo or "Togolese Republic" or Tonga or Tunisia or Turkey or Turkmenistan or Turkmen or Uganda or Ukraine or Uzbekistan or Uzbek or Vanuatu or "New Hebrides" or Venezuela or Vietnam or "Viet Nam" or "West Bank" or Yemen or Zambia or Zimbabwe)) OR ((Afghanistan or Albania or Algeria or Angola or Argentina or Armenia or Armenian or Azerbaijan or Bangladesh or Benin or Byelarus or Byelorussian or Belarus or Belorussian or Belorussia or Belize or Bhutan or Bolivia or Bosnia or Herzegovina or Hercegovina or Botswana or Brazil or Bulgaria or "Burkina Faso" or "Burkina Fasso" or "Upper Volta" or Burundi or Urundi or Cambodia or "Khmer Republic" or Kampuchea or Cameroon or Cameroons or Cameron or Camerons or "Cape Verde" or "Central African Republic" or Chad or China or Colombia or Comoros or "Comoro Islands" or Comores or Mayotte or Congo or Zaire or "Costa Rica" or "Cote d'Ivoire" or "Ivory Coast" or Cuba or Djibouti or "French Somaliland" or Dominica or "Dominican Republic" or "East Timor" or "East Timur" or "Timor Leste" or Ecuador or Egypt or "United Arab Republic" or "El Salvador" or Eritrea or Ethiopia or Fiji or Gabon or "Gabonese Republic" or Gambia or Gaza or "Georgia Republic" or "Georgian Republic" or Ghana or Grenada or Guatemala or Guinea or Guiana or Guyana or Haiti or Honduras or India or Maldives or Indonesia or Iran or Iraq or Jamaica or Jordan or Kazakhstan or Kazakh or Kenya or Kiribati or Korea or Kosovo or Kyrgyzstan or Kirghizia or "Kyrgyz Republic" or Kirghiz or Kirgizstan or "Lao PDR" or Laos or Lebanon or Lesotho or Basutoland or Liberia or Libya or Macedonia or Madagascar or "Malagasy Republic" or Malaysia or Malaya or Malay or Sabah or Sarawak or Malawi or Mali or "Marshall Islands" or Mauritania or Mauritius or "Agalega Islands" or Mexico or Micronesia or "Middle East" or Moldova or Moldovia or Moldovian or Mongolia or Montenegro or Morocco or Ifni or Mozambique or Myanmar or Myanma or Burma or Namibia or Nepal or "Netherlands Antilles" or Nicaragua or Niger or Nigeria or Muscat or Pakistan or Palau or Palestine or Panama or Paraguay or Peru or Philippines or Philipines or Phillipines or Phillippines or "Papua New Guinea" or Romania or Rumania or Roumania or Rwanda or Ruanda or "Saint Lucia" or "St Lucia" or "Saint Vincent" or "St Vincent" or Grenadines or Samoa or "Samoan Islands" or "Navigator Island" or "Navigator Islands" or "Sao Tome" or Senegal or Serbia or Montenegro or Seychelles or "Sierra Leone" or "Sri Lanka" or "Solomon Islands" or Somalia or Sudan or Suriname or Surinam or Swaziland or Eswatini or "South Africa" or Syria or Tajikistan or Tadzhikistan or Tadjikistan or Tadzhik or Tanzania or Thailand or Togo or "Togolese Republic" or Tonga or Tunisia or Turkey or Turkmenistan or Turkmen or Uganda or Ukraine or Uzbekistan or Uzbek or Vanuatu or "New Hebrides" or Venezuela or Vietnam or "Viet Nam" or "West Bank" or Yemen or Zambia or Zimbabwe))
- TI ((Africa or Asia or Caribbean or "West Indies" or "South America" or "Latin America" or "Central America")) OR AB ((Africa or Asia or Caribbean or "West Indies" or "South America" or "Latin America" or "Central America")) OR ((Africa or Asia or Caribbean or "West Indies" or "South America" or "Latin America" or "Central America"))
- DE "Developing Countries" OR DE "Argentina" OR DE "Aruba" OR DE "Bahamas" OR DE "Bahrain" OR DE "Barbados" OR DE "Belize" OR DE "Bermuda" OR DE "Bolivia" OR DE "Bonaire" OR DE "Brazil" OR DE "British Virgin Islands" OR DE "Brunei Darussalam" OR DE "Cameroon" OR DE "Cayman Islands" OR DE "Chile" OR DE "China" OR DE "Christmas Island" OR DE "Cocos Islands" OR DE "Colombia" OR DE "Congo" OR DE "Cook Islands" OR DE "Costa Rica" OR DE "Cote d'Ivoire" OR DE "Crozet Islands" OR DE "Cuba" OR DE "Curacao" OR DE "Cyprus" OR DE "Dominica" OR DE "Dominican Republic" OR DE "Easter Island" OR DE "Ecuador" OR DE "Egypt" OR DE "El Salvador" OR DE "Falkland Islands" OR DE "Federated States of Micronesia" OR DE "Fiji" OR DE "French Guiana" OR DE "Gabon" OR DE "Gambier Islands" OR DE "Ghana" OR DE "Grenada" OR DE "Guadeloupe" OR DE "Guam" OR DE "Guatemala" OR DE "Guyana" OR DE "Honduras" OR DE "India" OR DE "Indonesia" OR DE "Iran" OR DE "Iraq" OR DE "Jamaica" OR DE "Jordan" OR DE "Kenya" OR DE "Kerguelen Archipelago" OR DE "Korea Democratic People's Republic" OR DE "Korea Republic" OR DE "Kuwait" OR DE "Least Developed Countries" OR DE "Lebanon" OR DE "Libya" OR DE "Malaysia" OR DE "Marquesas Islands" OR DE "Marshall Islands" OR DE "Martinique" OR DE "Mauritius" OR DE "Mayotte" OR DE "Mexico" OR DE "Midway Islands" OR DE "Mongolia" OR DE "Montserrat" OR DE "Morocco" OR DE "Namibia" OR DE "New Britain" OR DE "New Caledonia" OR DE "New Ireland" OR DE "Nicaragua" OR DE "Nigeria" OR DE "Niue" OR DE "Northern Mariana Islands" OR DE "Oman" OR DE "Pakistan" OR DE "Panama" OR DE "Papua New Guinea" OR DE "Paraguay" OR DE "Peru" OR DE "Philippines" OR DE "Algeria" OR DE "Puerto Rico" OR DE "Qatar" OR DE "Reunion" OR DE "Saba" OR DE "Saint Helena" OR DE "Saint Kitts and Nevis" OR DE "Saint Lucia" OR DE "Saint Vincent and the Grenadines" OR DE "Saudi Arabia" OR DE "Senegal" OR DE "Seychelles" OR DE "Singapore" OR DE "South Africa" OR DE "Sri Lanka" OR DE "Suriname" OR DE "Swaziland" OR DE "Syria" OR DE "Tahiti" OR DE "Thailand" OR DE "Tokelau" OR DE "Tonga" OR DE "Angola" OR DE "Anguilla Island" OR DE "Trinidad and Tobago" OR DE "Tuamotu" OR DE "Tubuai Islands" OR DE "Tunisia" OR DE "Turkey" OR DE "Turks and Caicos Islands" OR DE "United Arab Emirates" OR DE "Uruguay" OR DE "Venezuela" OR DE "Vietnam" OR DE "Wallis and Futuna" OR DE "Western Sahara" OR DE "Zimbabwe" OR DE "Antigua and Barbuda"
- DE "Caribbean" OR DE "Bahamas" OR DE "Turks and Caicos Islands" OR DE "Antilles" OR DE "French West Indies" OR DE "Guadeloupe" OR DE "Martinique"
- DE "Pacific Islands" OR DE "Macquarie Island" OR DE "Melanesia" OR DE "Micronesia" OR DE "Norfolk Island" OR DE "Polynesia" OR DE "Wake Island" OR DE "French Polynesia" OR DE "Gambier Islands" OR DE "Marquesas Islands" OR DE "Society Islands" OR DE "Tuamotu" OR DE "Tubuai Islands" OR DE "Oceania" OR DE "Australasia" OR DE "Micronesia" OR DE "Polynesia"
- DE "South East Asia" OR DE "Brunei Darussalam" OR DE "Indochina" OR DE "Indonesia" OR DE "Malaysia" OR DE "Myanmar" OR DE "Philippines" OR DE "Singapore" OR DE "Taiwan" OR DE "Thailand" OR DE "West Asia" OR DE "Armenia" OR DE "Azerbaijan" OR DE "Iran" OR DE "Iraq" OR DE "Israel" OR DE "Jordan" OR DE "Kazakhstan" OR DE "Kyrgyzstan" OR DE "Lebanon" OR DE "Afghanistan" OR DE "Oman" OR DE "Palestine" OR DE "Persian Gulf States" OR DE "Republic of Georgia" OR DE "Saudi Arabia" OR DE "Syria" OR DE "Tajikistan" OR DE "Turkey" OR DE "Turkmenistan" OR DE "Uzbekistan" OR DE "Yemen" OR DE "East Asia" OR DE "China" OR DE "Japan" OR DE "Korea Democratic People's Republic" OR DE "Korea Republic" OR DE "Mongolia" OR DE "South Asia" OR DE "Bangladesh" OR DE "Bhutan" OR DE "India" OR DE "Nepal" OR DE "Pakistan" OR DE "Sri Lanka" OR DE "Central Asia" OR DE "Kazakhstan" OR DE "Kyrgyzstan" OR DE "Mongolia" OR DE "Afghanistan" OR DE "Tajikistan" OR DE "Turkmenistan" OR DE "Uzbekistan" OR DE "Xinjiang"
- DE "Mexico"
- DE "South America" OR DE "Argentina" OR DE "Bolivia" OR DE "Brazil" OR DE "Chile" OR DE "Colombia" OR DE "Ecuador" OR DE "Falkland Islands" OR DE "French Guiana" OR DE "Guyana" OR DE "Paraguay" OR DE "Peru" OR DE "Amazonia" OR DE "Suriname" OR DE "Uruguay" OR DE "Venezuela" OR DE "Latin America" OR DE "Argentina" OR DE "Bolivia" OR DE "Brazil" OR DE "Chile" OR DE "Colombia" OR DE "Costa Rica" OR DE "Cuba" OR DE "Dominican Republic" OR DE "Ecuador" OR DE "El Salvador" OR DE "Guatemala" OR DE "Honduras" OR DE "Mexico" OR DE "Nicaragua" OR DE "Panama" OR DE "Paraguay" OR DE "Peru" OR DE "Puerto Rico" OR DE "Uruguay" OR DE "Venezuela" OR DE "Central America" OR DE "Belize" OR DE "Costa Rica" OR DE "El Salvador" OR DE "Guatemala" OR DE "Honduras" OR DE "Nicaragua" OR DE "Panama"
- DE "Africa" OR DE "Francophone Africa" OR DE "Africa South of Sahara" OR DE "North Africa" OR DE "Portuguese Speaking Africa" OR DE "Anglophone Africa"

## APPENDIX C CODING TOOL

**Table:**

Study design	○ RCT ○ Quasi‐experimental study ○ Cluster‐quasi RCT ○ Systematic review ○ Regression discontinuity design ○ Controlled before and after study ○ Cost‐effectiveness analysis ○ Cost benefit analysis ○ Economic impact ○ Transport equity/justice
Publication status	○ Completed ○ Ongoing
Study methods	○ Difference in difference ○ Propensity score matching ○ Instrument variable/Heckmann selection ○ Multivariate/covariate adjusted analysis (e.g., ANCOVA analysis) ○ Bivariate analysis/comparison of means
Population	○ Rural ○ Urban ○ Both rural and urban
Region	○ East Asia & Pacific ○ Latin America & Caribbean ○ Middle East & North Africa ○ South Asia ○ Sub‐Saharan Africa ○ Europe & Central Asia
Intervention	○ Infrastructure ■ Roads ■ Rail, trams, monorail ■ Ports, shipping, and waterways ■ Civil aviation ○ Incentives ■ Roads ■ Rail, trams, monorail ■ Ports, shipping, and waterways ■ Civil aviation ○ Institutions (including regulations) ■ Roads ■ Rail, trams, monorail ■ Ports, shipping, and waterways
Outcome	○ Transport infrastructure, services and use ■ Infrastructure quantity ■ Infrastructure quality ■ Infrastructure services ■ Transport time and costs ■ Market access ■ Transport modality ■ Safe practices ○ Economic impact ■ Household income and poverty ■ Economic development ■ Employment and migration ■ Trade and tourism ■ Location, land use and prices ■ Displacement ○ Health and Education ■ Access to health facilities ■ Health outcomes ■ Access to education facilities ■ Education outcomes ○ Culture ■ Values, language and social cohesion ■ Cultural heritage ■ Cultural diversity ○ Environment ■ Air Quality ■ Noise pollution ■ Habitat destruction ○ Economic & equity analysis ■ Cost analysis inc CBA ■ Gender equity ■ Transport equity
Study type	○ Impact evaluations ○ Systematic review

**AMSTAR‐2 for systematic reviews**

1) Did the research questions and inclusion criteria for the review include the components of PICO?
  i) Yes
    (1) Population
    (2) Intervention
    (3) Comparator group
    (4) Outcome
    (5) Time frame for follow‐up (optional)
  ii) No
2) Did the report of the review contain an explicit statement that the review methods were established prior to the conduct of the review and did the report justify any significant deviations from the protocol?
  i) Yes: The authors state that they had a written protocol or guide that included ALL the following
    (1) Review question
    (2) Search strategy
    (3) Inclusion/exclusion criteria
    (4) A risk of bias assessment
    (5) A meta‐analysis/synthesis plan, if appropriate,
    (6) A plan for investigating causes of heterogeneity
    (7) Justification for any deviations from the protocol
  ii) Partial Yes: The authors state that they had a written protocol or guide that included ALL the following
    (1) Review question(s)
    (2) A search strategy
    (3) Inclusion/exclusion criteria
    (4) A risk of bias assessment
  iii) No
3) Did the review authors explain their selection of the study designs for inclusion in the review?
  i) Yes: If the review satisfy ONE of the following
    (1) Explanation for including only RCTs
    (2) OR Explanation for including only NRSI
    (3) OR Explanation for including both RCTs and NRSI
  ii) No
4) Did the review authors use a comprehensive literature search strategy?
  i) Yes: Should have all the following
    (1) Aearched at least two databases (relevant to research question)
    (2) Provided key word and/or search strategy
    (3) Justified publication restrictions (e.g., language)
    (4) Searched the reference lists/bibliographies of included studies
    (5) Searched trial/study registries
    (6) Included/consulted content experts in the field
    (7) Where relevant, searched for grey literature
    (8) Conducted search within 24 months of completion of the review
  ii) Partial yes: All the following
    (1) Searched at least two databases (relevant to research question)
    (2) Provided key word and/or search strategy
    (3) Justified publication restrictions (e.g., language)
  iii) No
5) Did the review authors perform study selection in duplicate?

i) Yes, either ONE of the following
  (1) At least two reviewers independently agreed on selection of eligible studies and achieved consensus on which studies to include
  (2) Two reviewers selected a sample of eligible studies and achieved good agreement (at least 80%), with the remainder selected by one reviewer.
ii) No

6) Did the review authors perform data extraction in duplicate?
  i) Yes: either ONE of the following
    (1) At least two reviewers achieved consensus on which data to extract from included studies
    (2) Two reviewers extracted data from a sample of eligible studies and achieved good agreement (at least 80%), with the remainder extracted by one reviewer
  ii) No
7) Did the review authors provide a list of excluded studies and justify the exclusions?
  i) Yes: if it includes the following
    (1) Provided a list of all potentially relevant studies that were read in full‐text form but excluded from the review
    (2) Justified the exclusion from the review of each potentially relevant study
  ii) Partial Yes if:
    (1) Provided a list of all potentially relevant studies that were read in full‐text form but excluded from the review
  iii) No
8) Did the review authors describe the included studies in adequate detail?
  i) Yes: should also have ALL the following
    (1) Described population in detail
    (2) Described intervention in detail (including doses where relevant)
    (3) Described comparator in detail (including doses where relevant)
    (4) Described study's setting
    (5) Timeframe for follow‐up
  ii) Partial Yes: should have the following
    (1) Described populations
    (2) Described interventions
    (3) Described comparators
    (4) Described outcomes
    (5) Described research designs
  iii) No
9) Did the review authors use a satisfactory technique for assessing the risk of bias (RoB) in individual studies that were included in the review?
  i) RCTs
    (1) Yes: must have assessed RoB from
      (a) Allocation sequence that was not truly random, and
      (b) Selection of the reported result from among multiple measurements or analyses of a specified outcome
    (2) Partial Yes: must have assessed RoB from
      (a) Unconcealed allocation, and
      (b) Lack of blinding of patients and assessors when assessing outcomes (unnecessary for objective outcomes such as all‐cause mortality)
    (3) No
  ii) NRSI
    (1) Yes: must also have assessed RoB from
      (a) Methods used to ascertain exposures and outcomes, and
      (b) Selection of the reported result from among multiple measurements or analyses of a specified outcome
    (2) Partial Yes: must have assessed RoB
      (a) From confounding, and
      (b) From selection bias
    (3) No
10) Did the review authors report on the sources of funding for the studies included in the review
  i) Yes: Must have reported on the sources of funding for individual studies included in the review. Note: Reporting that the reviewers looked for this information but it was not reported by study authors also qualifies
  ii) No
11) If meta‐analysis was performed did the review authors use appropriate methods for statistical combination of results?
  i) RCTs
    (1) Yes if
      (a) The authors justified combining the data in a meta‐analysis
      (b) AND they used an appropriate weighted technique to combine study results and adjusted for heterogeneity if present.
      (c) AND investigated the causes of any heterogeneity
    (2) No
    (3) No meta‐analysis conducted
  ii) For NRSI
    (1) Yes if
      (a) The authors justified combining the data in a meta‐analysis
      (b) AND they used an appropriate weighted technique to combine study results, adjusting for heterogeneity if present
      (c) AND they statistically combined effect estimates from NRSI that were adjusted for confounding, rather than combining raw data, or justified combining raw data when adjusted effect estimates were not available
      (d) AND they reported separate summary estimates for RCTs and NRSI separately when both were included in the review
    (2) No
    (3) No meta‐analysis conducted
12) If meta‐analysis was performed, did the review authors assess the potential impact of RoB in individual studies on the results of the meta‐analysis or other evidence synthesis?
  i) Yes if
    (1) Included only low risk of bias RCTs
    (2) OR, if the pooled estimate was based on RCTs and/or NRSI at variable RoB, the authors performed analyses to investigate possible impact of RoB on summary estimates of effect
  ii) No
  iii) No meta‐analysis conducted
13) Did the review authors account for RoB in individual studies when interpreting/discussing the results of the review?
  i) Yes if
    (1) Included only low risk of bias RCTs
    (2) OR, if RCTs with moderate or high RoB, or NRSI were included the review provided a discussion of the likely impact of RoB on the results
  ii) No
14) Did the review authors provide a satisfactory explanation for, and discussion of, any heterogeneity observed in the results of the review?
  i) Yes if
    (1) There was no significant heterogeneity in the results
    (2) OR if heterogeneity was present the authors performed an investigation of sources of any heterogeneity in the results and discussed the impact of this on the results of the review
  ii) No
15) If they performed quantitative synthesis did the review authors carry out an adequate investigation of publication bias (small study bias) and discuss its likely impact on the results of the review?
  i) Yes if
    (1) Performed graphical or statistical tests for publication bias and discussed the likelihood and magnitude of impact of publication bias
  ii) No
  iii) No meta‐analysis conducted
16) Did the review authors report any potential sources of conflict of interest, including any funding they received for conducting the review?
  i) Yes if
    (1) The authors reported no competing interests OR
    (2) The authors described their funding sources and how they managed potential conflicts of interest
  ii) No

a) Overall study quality
  i) High: No or one noncritical weakness *the systematic review provides an accurate and comprehensive summary of the results of the available studies that address the question of interest*
  ii) Moderate: More than one noncritical weakness
  iii) Low: One critical flaw* with or without noncritical weaknesses

## APPENDIX D DEFINITIONS OF INTERVENTIONS AND OUTCOME

**Table:**

Terms	Definition
Transport infrastructure, services and use—Transport infrastructure (e.g., roads, railways, ports, or airports), upgrading existing links and technology, or improving transport services, such as public bus services. Services available, introduced and usage of the available transport infrastructure.	
Infrastructure quantity	Infrastructure increase or growth
Infrastructure quality (inc. safety assessment)	Quality of the available infrastructure (Road quality)
Infrastructure services	Logistics—transportation of the agricultural products, goods and other materials.
Transport time or costs (inc. congestion and VOC)	Access to transport infrastructure, travel time, time taken to access the available transport infrastructure, frequency of service, connectivity, travel cost and Congestion.
	Congestion in transport is a major problem in both developed and developing countries involving high opportunity costs
Market access	Access to market by the population and it also include the access by the enterprises or farmers to sell their goods in the market.
Transport modality (inc. car ownership)	Modes of transportation and it include ownership (Car)
Safe practices	Safe practices such as speed limits, use of helmets and other practices.
Economic Impact—Economic impact analysis is an exercise to determine how a transport intervention project or policy affects the amount and type of economic activity in a region. Provision of transport as a service to reduce poverty by increasing economic efficiency and enhancing opportunities. Transport allows people to reach out to job or its effects on employment opportunities and migration.	
Household income and poverty	Increase in household income, Poverty
Economic development	Enterprise development (profitability), GDP and Agricultural production
Employment and migration	Increase in employment opportunities
	Road accessibility has impact on population movements.
Trade and tourism	Transfer of goods and services, trade activities and tourism development and affects.
Location (land use) and prices	Locations of the firm or the household. Effects on the prices of the property.
Displacement	Displacement of the population due to transport infrastructure. (construction, other infrastructure development projects)
Health and education—Transport can affect health, both positive (access to health services, higher income, availability of more diversified diet, etc.) and negative (road traffic injuries, air pollution, and spreading disease).	
Education also affected by the transport, it gives access to educational facilities and lack of transport infrastructures affect the educational status of the population.	
Access to health facilities	Health facilities—Health centres (Primary and secondary) and Emergency services (Obstetric)
Health outcomes	Health related outcomes—Improved health status, improvement in health conditions disease.
	Spread of disease—Transport systems can also help spread infectious diseases, such as the recent Ebola epidemic (World Bank, 2014) and COVID‐19
	Road traffic injuries—Rise in fatalities and road injuries especially in LMICs due to poor quality of roads and road safety regulations
Access to education facilities	Education facilities such as schools, college, or vocational centres.
Education outcomes	Educational status—School enrolment, attendance rate, dropout rates.
Culture—Cultural effects, both positive and negative consequences of increased mobility within and between nations.	
Values, language and social cohesion	Effects on the social cohesion, values of the population (Due to forced displacement and migration)
Cultural heritage	
Cultural diversity	Different cultural, its diversity
Environment—Transport system may also disturb ecosystem through deforestation, biodiversity loss, pollution, road kill, and blocking of seasonal migration patterns of wildlife	
Air quality	Air pollution caused by vehicle emissions, from increased traffic volumes.
Noise pollution	Sounds of Vehicle and Industrial areas (Transport Hub)
Habitat destruction	Habitat loss and habitat reduction due to improved transport infrastructure
Economic and equity analysis	
Cost Analysis inc CBA	CBA—Cost‐benefit/benefit‐cost analysis is an exercise to determine the social welfare effects of transport sector interventions in comparison to costs.
Gender equity	Promoting women travellers, provision or benefits to women in transport infrastructure, Gender promotion.
Transport equity^8^	Transportation equity or justice usually refers to the fairness with which the impacts of transportation such as benefits and costs are distributed. Horizontal equity, also called fairness and egalitarianism, is concerned with the distribution of impacts between individuals and groups considered equal in ability and need; vertical equity is concerned with the distribution of impacts between individuals and groups that differ in abilities and needs, for example, by income or social class (also called social justice, environmental justice and social inclusion) or in transportation ability and need otherwise known as universal design

8 Transportation equity or justice usually refers to the fairness with which the impacts of transportation such as benefits and costs are distributed. Horizontal equity, also called fairness and egalitarianism, is concerned with the distribution of impacts between individuals and groups considered equal in ability and need; vertical equity is concerned with the distribution of impacts between individuals and groups that differ in abilities and needs, for example, by income or social class (also called social justice, environmental justice and social inclusion) or in transportation ability and need otherwise known as universal design (Litman, 2018).
